# Prophylactic gastrostomy in locally advanced head and neck cancer: results of a national survey among radiation oncologists

**DOI:** 10.1186/s12885-021-08348-9

**Published:** 2021-06-02

**Authors:** Tatiana Dragan, Fréderic Duprez, André Van Gossum, Akos Gulyban, Sylvie Beauvois, Antoine Digonnet, Yassine Lalami, Dirk Van Gestel

**Affiliations:** 1 Department of Radiation Oncology (Head and Neck Unit), Institut Jules Bordet, Université Libre de Bruxelles, 1 rue Héger Bordet – 1000 Bruxelles, Brussels, Belgium; 2 Department of Radiotherapy-Oncology, Universitair Ziekenhuis Gent, Ghent, Belgium; 3 Consultant at the Department of Gastroenterology and Clinical Nutrition, Hopital Erasme and Institut Jules Bordet, Brussels, Belgium; 4 Medical Physics Department, Institut Jules Bordet, Brussels, Belgium; 5 Department of Head and Neck Surgery, Institut Jules Bordet, Université Libre de Bruxelles, Brussels, Belgium; 6 Medical Oncology Clinic, Institut Jules Bordet, Université Libre de Bruxelles, Brussels, Belgium

**Keywords:** Endoscopic gastrostomy, Head and neck cancer, Radiotherapy, Survey

## Abstract

**Background:**

Nutritional complications in patients with locally advanced head and neck cancer (LA-HNC) treated by concurrent chemoradiotherapy (CCRT) often lead to placement of a prophylactic gastrostomy (PG) tube, while indication lacks harmonization. Our aim was to explore the current PG tube utilization among Belgian radiation oncology centers.

**Methods:**

A survey was distributed to all 24 Belgian Radiation oncology departments, with questions about the number of patient treated per year, whether the PG indication is discussed at the multidisciplinary board, placement technique, time of starting nutrition and removal, its impact on swallowing function and importance of clinical factors. For the latter Relative Importance and Discordance Indexes were calculated to describe the ranking and agreement.

**Results:**

All 24 centers submitted the questionnaire. Twenty three treat more than 20 head and neck (HNC) patients per year, while four (1 in 21–50; 3 in 51–100) are not discussing the gastrostomy tube indication at the multidisciplinary board. For the latter, endoscopic placement (68%) is the dominant technique, followed by the radiologic (16%) and laparoscopic (16%) methods. Seventy-five percent start the enteral nutrition when clinically indicated, 17% immediately and 8% from the start of radiotherapy. Majority of specialists (19/24) keep the gastrostomy tube until the patient assume an adequate oral feeding. Fifteen centres are considering PG decrease swallowing function. Regarding factors and their importance in the decision for the PG, foreseen irradiated volume reached highest importance, followed by ‘anatomical site’, ‘patients’ choice’ and ‘postoperative versus definitive’ and ‘local expertise’, with decreasing importance respectively. Disagreement indexes showed moderate variation.

**Conclusions:**

The use of a PG tube for LAHNC patients treated by CCRT shows disparity at national level. Prospective studies are needed to ensure proper indication of this supportive measure.

## Background

Chemotherapy combined with Intensity modulated radiation therapy is the standard of care in organ preserving, definitive therapy for locally advanced head and neck cancer (LA-HNC) patients [1, 2]. Advances such as accelerated radiotherapy and concurrent chemoradiation (CCRT) have improved the tumor related prognosis [3–6]. The addition of cisplatin-based chemotherapy improves both locoregional control and overall survival (OS) when compared with radiotherapy alone, but at the price of a substantial increase in severe toxicity [7]. Toxicities which occur during CCRT like mucositis, swallowing disorders, xerostomia and taste distortion often impact on oral intake with an increased risk of malnutrition, dehydration, low recovery of symptoms and weight loss. This may affect the treatment compliance with a detrimental impact on disease control [8]. Furthermore, a poor nutritional status before and during treatment may be associated with a worse clinical outcome and quality of life [9–11]. The gastrostomy feeding tube is the most common approach to improve nutrition in HNC patients undergoing CCRT. In patients without dysphagia before the initiation of CCRT there is no consensus regarding the optimal timing for the gastrostomy tube placement [12]. Certain centers prefer a prophylactic placement, others a reactive, i.e. when clinically indicated. The risk factors to determine who will benefit from a PG tube are not well defined and the impact on late swallowing function is unclear [13]. Late dysphagia has previously been found a very frequent complaint (≈ 43%) and one of the most relevant side-effects after HNC treatment, with an impact on quality of life which is even more important than the impact of xerostomia [7, 14]. In some situations (13%) this might even lead to long term gastrostomy feeding tube use (> 2 years after treatment) [7]. The systematic intervention of a speech therapist, on the other hand, can prevent late dysphagia in some and lower its intensity in others [15].

We conducted this survey in order to explore the current practice on PG tubes in Belgian radiation oncology departments by collecting general information and expertise in the treatment of HNC patients and to see whether there is a national consensus.

## Methods

The survey contained nine questions. The following issues were assessed: the importance of a multidisciplinary board in taking decisions, the decisive factors for the indication of PG tube placement, the techniques of gastrostomy tube placement available in each centre, the nutritional management after PG tube placement and the specialist’s opinion on the PG’s impact on late swallowing function after CCRT (Table 1).

**Table 1:** Questions of the survey

1. Speciality	
2. Instutution	
3. Is the indication for prophylactic gastrostomy discussed at the multidisciplinary board?	Yes/No
4. How many patients do you irradiate per year in your center	0/0–20/21–50/51–100/> 100
5. In case of a Locally Advanced Head and Neck Cancer (LAHNC) patient, without dysphagia nor contraindications for gastrostomy, with normal nutritional status who will undergo chemo-radiation, to which degree the following factors would influence your decision: a) the foreseen irradiated volume of oral/oropharyngeal mucosa, constrictor muscles and oesophagus?; b) the anatomical site of the tumour?; c) the postoperative versus curative setting?; d) the patient’s choice?; e) the expertise of your centre in gastrostomy placement?	Not at all important/Slightly Important/Important/Fairly Important/Very Important
6. In your centre which technique is used to place gastrostomies (you can select more than one if necessary)	Endoscopic/Radiologic/Laparoscopic/Gastrostomies are not used/Other
7. When do you start enteral nutrition via prophylactic gastrostomy?	Immediately following the prophylactic gastrostomy placement/from the start of radiotherapy/Later, when clinically indicated/Other (free text)
8. When do you remove the prophylactic gastrostomy?	At the end of chemo-radiation/When the patient is able to assume an adequate oral feeding/In case of complete locoregional remission at the first evaluation post chemo-radiation/Other, please specify (free text)
9. In your opinion, could the use of a prophylactic gastrostomy have a negative impact on swallowing function after chemo-radiation?	Yes/No/No opinion

All questions were defined and approved in consensus by a multidisciplinary team of specialists involved in the treatment of the HNC patients: specialised clinical nurse, speech pathologist, dietitian, gastroenterologist, radiation oncologist, medical oncologist and head and neck surgeon. The survey was designed by the web application Survey Monkey and was sent via email to the 24 radiation oncologists specialised in the HNC treatment from all 24 primary Belgian radiotherapy departments. An initial email with a brief explanation of the study and an invitation to complete the survey was sent in August, 2019, and a reminder email for those who had not completed the survey was sent in January, 2020. Respondents had to answer all questions in the survey before they could submit it.

### Data analysis and statistics

Survey data was analysed with descriptive statistics using Microsoft Excel (version 2016, Redmond, Washington, USA). Results are reported in absolute and relative frequencies. To analyse the data under question 4, the responses were summed so that each clinical factor received a score for the number of respondents at each degree of importance (1 being very important to 5 being not at all important). We used the Relative Importance Index:

$$ \mathrm{Relative}\ \mathrm{Importance}\ \mathrm{Index}=\frac{\sum w}{A\cdot N}=\frac{5\cdot {n}_5+4\cdot {n}_4+3\cdot {n}_3+2\cdot {n}_2+1\cdot {n}_1}{5\cdot N} $$

Where ***w*** is the weighting given to each factor (in this case from 1 to 5), *n*_*x*_ represents the number of respondents for importance *x*, ***N*** is the total number of replies and *A* corresponds to the highest score (in our case *A = 5*), resulting the RII between 0 and 1. These values then were used to determine the rank (from 1 to 5, for each question).

To assess the disagreement we introduced the Relative Discordance Index (RDI) as follows:

$$ \mathrm{Relative}\ \mathrm{Discordance}\ \mathrm{Index}=\frac{\sum r}{A\cdot N}=\frac{\left|{r}_5-5\right|\cdot {n}_5+\left|{r}_4-4\right|\cdot {n}_4+\left|{r}_3-3\right|\cdot {n}_3+\left|{r}_2-2\right|\cdot {n}_2+\left|{r}_1-1\right|\cdot {n}_1}{5\cdot N} $$

where ***r*** is the weighting discordance for each factor (in this case from 1 to 5), ***r***_***x***_ and ***n***_***x***_ represents the rank and number of respondents for importance *x*, ***N*** is the total number of replies and *A* corresponds to the highest score (in our case *A = 5*), leading to an agreement ranking by assigning from 1 to 5 corresponding to the increase in RDI values (best agreement has lowest RDI).

## Results

**Fig. 1:**
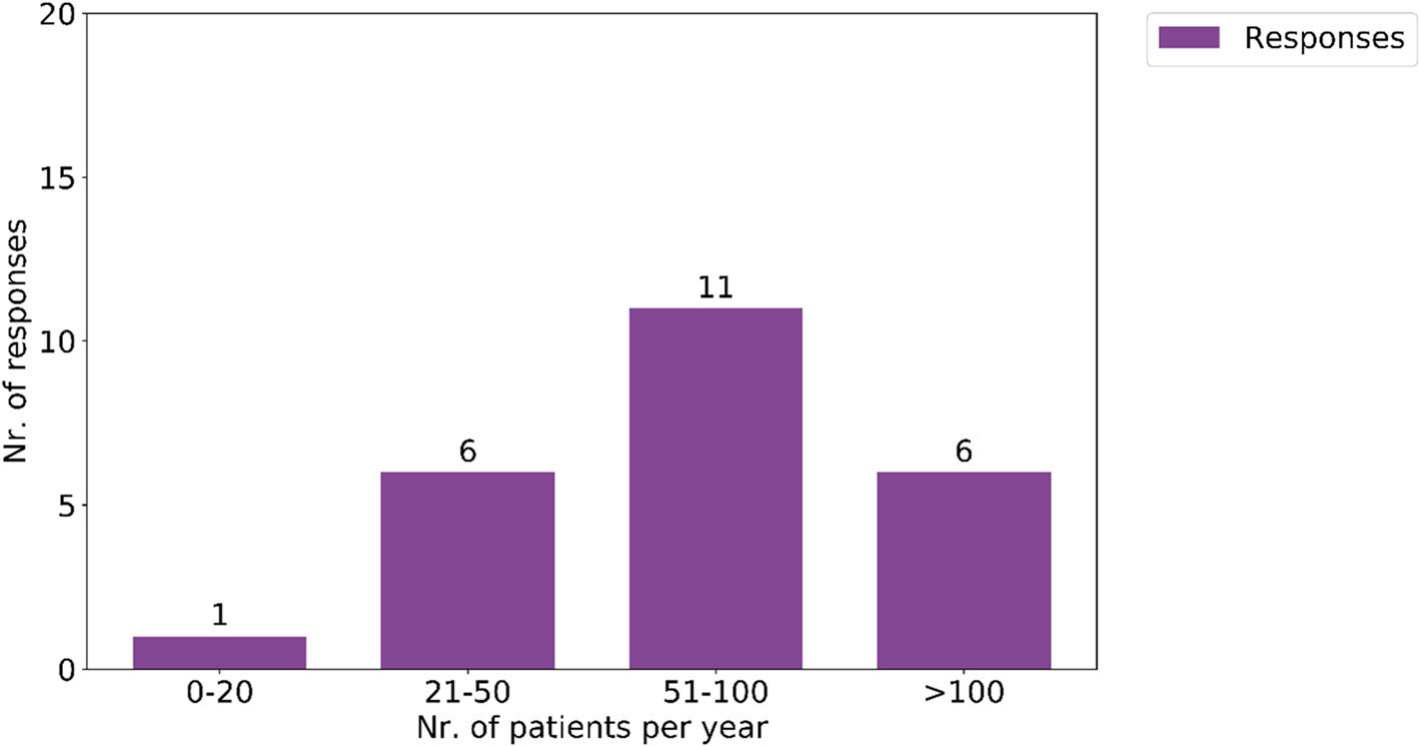
Number of HNC patients treated in each radiation oncology department per year

All Belgian Radiation Oncology departments filled out the questionnaire. Figure 1 illustrates the number of HNC patients treated in each radiation oncology department per year.

**Fig. 2:**
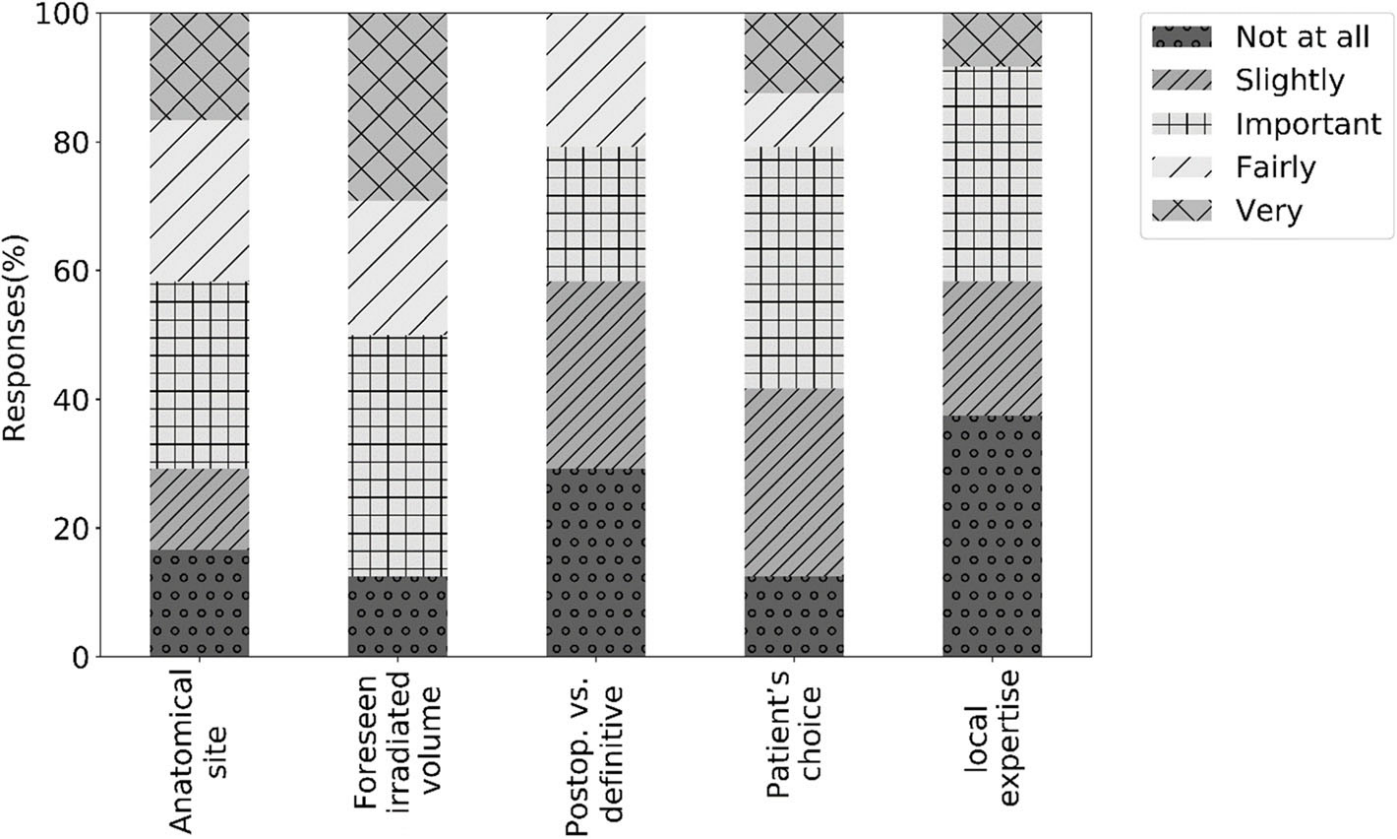
Question 5: Five factors and their importance in the decision for the prophylactic gastrostomy

Twenty centres (83%) stated the indication for gastrostomy tube placement to be discussed at the multidisciplinary board, four centres do not discuss it multidisciplinary. The most commonly used technique is endoscopic placement (68%), followed by the radiologic (16%) and laparoscopic (16%) method. Twenty nine percent stated that more than one option is used in their centre. Figure 2 shows five factors and their importance in the decision for the PG in case of a LA-HNC patient, without dysphagia nor contraindications for gastrostomy, with normal nutritional status who is about to undergo CCRT.

The results of the Relative Importance Index, Relative Discordance Index and the corresponding ranking of importance and agreement are presented in Table 2.

**Table 2:** Ranking of importance and agreement using RII and RDI (based on question nr.5)

	anatomical site	foreseen irradiated volume	postoperative vs. definitive	patient’s choice	local expertise
Relative Importance Index	0.58	0.49	0.73	0.64	0.76
Importance Rank (1 = most, 5 = least)	2	1	4	3	5
Relative Discordance Index	0.24	0.29	0.18	0.18	0.24
Agreement Rank (1 = best, 5 = least)	3	5	1	1	3

‘Foreseen irradiated volume’ was considered the most important, even with the highest disagreement, followed by ‘anatomical site’ with moderate agreement. ‘Patients’ choice’ and ‘postoperative versus definitive settings’ showed the best agreement at important and slightly important scores, while ‘local expertise’ was considered least important with moderate agreement. When considering the start of enteral nutrition via PG, 75% (18/24) responded ‘when clinically indicated’, 17% (4/24) ‘immediately following the PG placement’ and 8% (2/24) ‘from the start of radiotherapy’. Seventy nine percent of specialists (19/24) would keep the gastrostomy tube until the patient is able to assume an adequate oral feeding, 4% (1/24) awaits the patient to be considered in complete remission while 17% (4/24) expect both criteria to be fulfilled. Sixty three percent (15/24) stated that in their opinion the use of the PG could have a negative impact on the swallowing function after CCRT; 33% (8/24) did not expect impact and 4% (1/24) did not had an opinion.

## Discussion

The use of a PG tube to prevent malnutrition in HNC patients receiving definitive CCRT has gained a lot of attention in recent literature; however, the appropriate guidelines in clinical practice have not been established yet. This survey reporting on the Belgian HNC radiation oncologists’ current clinical practice and their opinion on whether and when to consider a PG tube had a 100% response rate.

Ninety six percent of centers reported to yearly irradiate more than 20 HNC patients and 71% even treat more than 50. Facility volume improves a variety of clinical processes, including access to supportive care such as pain management, swallow/speech therapy and nutrition that increase the probability of treatment completion, minimize the likelihood of treatment interruptions, and mitigate morbidity. There is an emergent body of evidence that patients with HNC who are treated at high-volume centers also have better outcomes [16–19]. Within a randomized trial of the Radiation Therapy Oncology Group (RTOG 0129), which compared cisplatin concurrent with standard versus accelerated fractionation radiotherapy Wuthrick et al. found the 5-year OS rate to be 69.1% vs 51.0% (*p* = .002), respectively, for patients treated at historically low- vs high-accruing centers [20]. In 2019, the Belgian Health Care Knowledge Centre (KCE) published an evaluation report on the quality of care in HNC patients in Belgian hospitals according to quality indicators and objectives defined by a panel of experts. According to this KCE report, 9175 head and neck squamous cell carcinoma (SCC) were treated in 99 different centers during the 6 y study period. It was noted that the median survival of patients treated in high-volume centers (hospitals treating more than 20 patients per year) was 1.1 year longer than their peers treated in low-volume centers (5.1 versus 4.0 years) [21]. Regarding radiotherapy volume, 4539 head and neck squamous cell carcinoma (HNSCC) were treated in Belgian radiotherapy (RT) centers between 2009 and 2014. The median RT center volume was 169 patients over the 6 y period (i.e. 28 patients per year) with a quarter of the centers treating less than 17 patients per year. There was no statistically significant association between RT center volume and overall survival among patients with HNSCC (*p* = 0.61). Assuming that our respondents’ answers are in agreement with the clinical practice, 96% of Belgian radiotherapy centers are in line with this quality indicator of offering personalized care and treatment to more than 20 HNC patients per year. The difference with KCE numbers of treated patients in RT departments can be explained by the KCE selection criteria which included only first treatments for SCC of the oral cavity, oropharynx, hypopharynx and larynx (nasal cavities, thyroid and salivary glands excluded) while 3287 patients (26%) with multiple synchronous tumors were left out of the analysis.

We found 83% of all gastrostomy tube indications to be discussed in a HNC dedicated multidisciplinary board. In the absence of a golden standard, the role of the interdisciplinary team is crucial to assess for each case the appropriateness of a nutritional intervention [22]. A multidisciplinary approach provides more accurate treatment recommendations, communication, reinforces cooperation, coordination and adherence to clinical guidelines [22, 23]. The combination of a HNC multidisciplinary expert team in a high volume referral cancer centre is considered an important indicator of quality of care for HNC and associated with better therapeutic decisions [24].

The most interesting results from our survey include the different factors and their importance in the decision for the PG in case of a HNC patient. Predicting which patient will benefit from PG is challenging. However, we were able to evaluate a number of factors that may correlate with the development of swallowing dysfunction during CRT. The foreseen irradiated mucosal volume, followed by anatomical site has been considered most important by the respondents. It is clear that the irradiated mucosal volume is different in function of anatomical site and lymph node involvement. Many studies have demonstrated a relationship between the dose received by anatomical structures involved in swallowing (e.g. the superior pharyngeal constrictor muscle) and radiation induced acute and late dysphagia [25–31]. Subsequently, several studies are currently focusing on reducing the elective radiation dose and the irradiated volume in order to decrease acute and late swallowing dysfunction [32–35]. Langendijk et al. developed a predictive model to identify patients at high risk of radio-induced dysphagia [14]. Advanced tumour stage (T3-T4), oropharyngeal and nasopharyngeal tumour site, primary and bilateral neck irradiation, weight loss at baseline, and treatment modality (accelerated RT or CCRT) were identified as independent factors predicting swallowing dysfunction. In Belgium, such studies are focussing on reduction of the elective dose and on volume individualisation of the prophylactic nodal target irradiated zone using the identification of the sentinel node [33, 35].

Another factor we evaluated is the patient’s choice. Despite the growing interest in supporting the patient’s participation in clinical decisions, there is no evidence to guide clinicians regarding the level of patients’ involvement in the decision-making process. Patient’s preference for involvement may vary between those preferring to take part on their own treatment, to those who prefer to leave treatment decisions to their medical team, largely as patients report lacking the specialized knowledge needed to make treatment decisions [36].

Swallowing dysfunction also has a significant impact on health-related quality of life, even more than xerostomia, as reported by patients [14]. Prophylactic gastrostomy tube may also negatively affect the psychological status of the patients as it may interfere with family life, intimate relationships and social activities [37]. However, a recent systematic review of the effect on enteral tube feeding on health related quality of life suggests PG placement to be effective in improving quality of life for patients with HNC cancers treated with CCRT [38].

The respondents accorded moderate importance in the decision making regarding PG tube to the sort of multimodality treatment, more precisely the postoperative versus definitive setting. Surgery before radiotherapy and extent of reconstruction appear to be important factors to develop swallowing problems during postoperative treatment [39–41]. Finally, local expertise and available techniques are important in the planning of the PG tube. Different techniques of gastrostomy placement are available across the Belgian radiotherapy centres, with endoscopic placement being the most commonly used (68%). There are, however, no randomised trials comparing these different techniques, hence current evidence is only based on retrospective and non-randomized controlled studies [42]. It is recommended to base the choice of the technique on indications and contraindications, local experience and the available techniques [43]. Complications related to the percutaneous endoscopic gastrostomy (PEG) tube placement by different techniques are quite rare and range from minor infections and bleeding to peritonitis [44]. One of the most serious complications is abdominal wall metastasis following PEG placement. This risk is correlated with advanced tumour stage, tumour biology and the technique [45]. As such, the “Pull” technique instead of the “Push” technique was identified as a risk factor in a large retrospective study with 777 HNC patients where the incidence of abdominal wall metastasis was 0.64% [46].

Regarding optimal starting of the enteral nutrition, the majority of respondents opted to start nutritional support when clinically indicated, more specifically in case of deterioration of swallowing or nutritional status including weight loss. As the CCRT side effects usually start around the second or third week of treatment, patients could continue oral food intake these first couple of weeks of radiotherapy. The benefit of the maintenance of oral intake was demonstrated by Hutcheson et al. who retrospectively analysed swallowing outcome in 497 HNC patients treated with CCRT. Maintenance of oral intake throughout treatment was associated with better long term swallowing function and less long term gastrostomy dependency [15]. Brown et al. conducted a randomized controlled study comparing early versus postponed feeding nutrition (i.e. when clinically indicated) in HNC patients with PG tube placement. They concluded that early use of the PG tube did not result in an increase in long term dependency. However, swallowing outcome measurements were not included in this study [47]. As there are no general guidelines on when to remove the gastrostomy the majority of survey respondents opted to wait until the patient is able to assume an adequate oral feeding. This is defined as the moment when the patients will be able to resume their protein and caloric need (at least 30 kcal/kg/day and 1.2 g protein/kg/day) by mouth prior to tube removal. Alternatively, it is defendable to wait for the first follow up exams to ensure that no further salvage therapy is required. Salvage treatment such as neck dissection could increase the risk of PEG tube dependency in HNC patients [48].

One of the most interesting results from the data presented is the disparity with regard to the influence of PG tube on swallowing outcome. This is consistent with published evidence on the possible negative effect of PG tube placement on long-term swallowing function [49–51]. Shaw et al. conducted a systematic review on this subject and concluded to a lack of consensus in literature regarding the impact on late swallowing function of the use of a PG [13]. We found the main debate to be about the importance of maintaining adequate nutrition during treatment versus maintaining swallowing function.

In our institution, we apply locally agreed recommendations in relation to the PG placement and the specialized/dedicated dietitian is part of the multidisciplinary team for HNC patients. Treatment intensification using multimodality approaches, such as surgery with adjuvant (chemo-) radiotherapy, accelerated radiotherapy, concomitant chemotherapy, etc., increases the cumulated toxicity, making a decline in nutritional status of HNC patients more probable. Therefore, many patients with newly diagnosed LA-HNC (stage III-IV) are offered a PG tube before the start of treatment even if initially they don’t have significant dysphagia. As such we do think that for those locally advanced tumors most treatments beyond radiotherapy alone deserve at least the consideration of a PG. This is especially the case when a lot of the swallowing structures will be irradiated or when the total amount of irradiated mucosa is judged important, i.e. in case of bilateral neck irradiation. Furthermore, the expected dose to the oral cavity is also considered an important risk factor and patients with locally advanced oral cavity tumors are referred for PG even in case of an exclusive radiotherapy. Prior surgery in LA-HNC, especially oral cavity and laryngeal cancer, is considered an important risk factor for deterioration of the nutritional status before, and after adjuvant radiotherapy, hence a PG is systematically recommended in our institution. At the same time some Belgian institutions do not recommend a PG tube in a patient without dysphagia and with normal nutritional status because of the potential risks related to the PG placement, such as infections, delayed systemic treatment administration, tumor dissemination and unknown impact on long term swallowing outcomes. Reactive PG tube placement could allow patients to have a shorter duration of usage while maintaining oral nutrition and may be less late dysphagia. The choice for PG is generally based on the experience of each institution and is not based on any high level evidence. In order to answer the question of which modality to provide better functional outcome for the patients and to make some evidence based recommendations we are actually performing a prospective randomized trial to assess patient reported outcomes in terms of swallowing and quality of life after prophylactic versus reactive gastrostomy tube placement in advanced oropharyngeal cancer patients treated with definitive CCRT.

The findings of this survey have to be seen in light of some limitations. The first and most important is the subjective matter given the survey is opinion based. The questions were designed with the multidisciplinary team for clarity and reliability however they have not been tested before being sent to the participants. Additionally, only a single national speciality was surveyed (radiation oncology), while the opinions of head and neck surgeons, medical oncologists and other physicians who care for HNC patients being clearly important, they were outside of the scope of this survey.

## Conclusions

This survey confirms the decision making for the placement and use of a PG tube in the context of LAHNC patients undergoing CCRT to be a complex process with a widely variable clinical practice. There is an imperative necessity for standardisation of recommendations and clinical guidelines. Further solid research is essential to support a better, evidence based clinical practice.

## Data Availability

All data generated or analysed during this survey are included in this published article.

## Abbreviations

CCRT: Concurrent chemoradiotherapy
HNC: Head and neck cancer
HNSCC: Head and neck squamous cell carcinoma
KCE: Belgian Health Care Knowledge Centre
LA-HNC: Locally advanced head and neck cancer
OS: Overall survival
PEG: Percutaneous endoscopic gastrostomy
PG: Prophylactic gastrostomy
RDI: Relative Discordance Index
RII: Relative Importance Index
RT: Radiotherapy
RTOG: Radiation Therapy Oncology Group
